# Conflicts in Mitochondrial Phylogenomics of Branchiopoda, with the First Complete Mitogenome of Laevicaudata (Crustacea: Branchiopoda)

**DOI:** 10.3390/cimb45020054

**Published:** 2023-01-18

**Authors:** Xiaoyan Sun, Jinhui Cheng

**Affiliations:** State Key Laboratory of Palaeobiology and Stratigraphy, Nanjing Institute of Geology and Palaeontology and Center for Excellence in Life and Palaeoenvironment, Chinese Academy of Sciences, 39 Beijing Eastroad, Nanjing 210008, China

**Keywords:** Laevicaudata, mitochondrial genome, compositional heterogeneity, phylogenetic signal, model violation

## Abstract

Conflicting phylogenetic signals are pervasive across genomes. The potential impact of such systematic biases may be reduced by phylogenetic approaches accommodating for heterogeneity or by the exclusive use of homoplastic sites in the datasets. Here, we present the complete mitogenome of *Lynceus grossipedia* as the first representative of the suborder Laevicaudata. We employed a phylogenomic approach on the mitogenomic datasets representing all major branchiopod groups to identify the presence of conflicts and concordance across the phylogeny. We found pervasive phylogenetic conflicts at the base of Diplostraca. The homogeneity of the substitution pattern tests and posterior predictive tests revealed a high degree of compositional heterogeneity among branchiopod mitogenomes at both the nucleotide and amino acid levels, which biased the phylogenetic inference. Our results suggest that Laevicaudata as the basal clade of Phyllopoda was most likely an artifact caused by compositional heterogeneity and conflicting phylogenetic signal. We demonstrated that the exclusive use of homoplastic site methods combining the application of site-heterogeneous models produced correct phylogenetic estimates of the higher-level relationships among branchiopods.

## 1. Introduction

With an increasing number of mitogenomes being sequenced and various methodological advances, mitogenomic data have been successfully utilized to improve phylogenetic reconstructions across a wide range of taxa [1,2,3]. The large amount of available mitogenomic data has reduced the stochastic error (sampling error) on phylogenetic inference. Nevertheless, deep relationships between Arthropoda at the interordinal or intraordinal level have not been fully resolved, resulting in topologies with high support frequently conflicting with morphological and nuclear phylogenies [4,5,6]. Such strong support but incorrect phylogenies represent systematic errors which can be traced back to homoplastic characteristics in datasets and model violations [7,8]. Substitutional saturation was the most frequently discussed cause of homoplasy in nucleotide gene data [9]. Most substitution models assume compositional homogeneity (stationary), but nucleotide and protein sequences might also exhibit nonstationarity, which strongly violates the assumptions of the stationary models [10,11]. The most common source of model violations are compositional heterogeneity and rate heterogeneity among lineages [12,13,14].

Suborder Laevicaudata Linder, 1945 (Branchiopoda: Diplostraca), or smooth clam shrimp, is a unique group of essentially benthic micro-crustaceans. Among branchiopods, Laevicaudata can be recognized by a usual body length less than 7 mm, a bivalved carapce, a proportionally large head, bearing a row of large teeth on the mandibular molar surface and having laminae abdominalis supporting egg clusters [15,16,17,18,19]. Laevicaudata currently comprises about 42 valid species worldwide except in Antarctica [15,16,18,20,21,22,23,24]. Laevicaudata, including only one family, Lynceidae Baird, 1845, is composed of three genera: *Lynceus* Müller, 1776; *Lynceiopsis* Daday, 1912; and *Paralimnetis* Gurney, 1931. They are distinguished by the shape and size of male claspers, characteristic modified second thoracopods, and rostrum [15,16,17,20], and *Lynceus* represents nearly 90% of all laevicaudatan species diversity [17,18,25].

The higher-level relationships of Branchiopoda are well resolved based on both morphological and molecular data [26,27,28,29,30,31,32]. The class Branchiopoda is divided into two superorders, Anostraca and Phyllopoda, and three extant orders, Anostraca, Notostraca and Diplostraca. Laevicaudata and Onychocaudata (Spinicaudata, Cyclestherida and Cladocera) are sister clades, forming the order Diplostraca, which, together with the order Notostraca, belongs to the superorder Phyllopoda [26,27,28]. It is widely accepted that Laevicaudata and Onychocaudata form a well-defined monophyletic order called Diplostraca [26,27,28,29,30]. Mitogenomes have substantially aided in estimating phylogenetic relationships within clades of Branchiopoda such as Anostraca, Notostraca and Cladocera [33,34,35,36], but limited success has been achieved in resolving the deep relationship between Laevicaudata and Onychocaudata. The large amount of available mitogenomic data provides a high phylogenetic resolution of the relationships among Cladocera. However, phylogenetic estimates thus far have resulted in strong support but incorrect phylogenies for Diplostraca, even using a site-heterogeneous mixture model [37], which might indicate a systematic bias arising from model violation.

In order to investigate the phylogenetic signal contained in the mitogenomes for major groups of Branchiopoda, we sequenced and annotated the complete mitogenome of *L. grossipedia* (Lynceidae) as the first complete mitogenome of the suborder Laevicaudata. The aim of this study is to account for the heterogeneity of sequences and dissect phylogenetic signals in the mitogenomic dataset, coupling them with available mitogenomes of Branchiopoda from GenBank (http://www.ncbi.nlm.nih.gov, accessed on 28 September 2022). We evaluated three methods for diminishing the non-phylogenetic signal concerning their effectiveness in reducing model violations and their influence on the phylogenetic reconstruction. We also compared the results of phylogenetic reconstruction with different approaches based on different datasets. Finally, we conducted four-cluster likelihood mapping analyses (FcLM) to evaluate and visualize the phylogenetic signal in each dataset.

Herein, using the higher-level relationships described above as correct topology, we presented the pervasiveness of phylogenetic conflicts at the base of Diplostraca. The results uncovered hitherto unrecognized nonphylogenetic signals as the artifactual origin of the conflicting topologies. The verification methods explicitly taking systematic bias into consideration consistently supported the monophyly of the Diplostraca.

## 2. Materials and Methods

### 2.1. Sample Collection and DNA Extraction

*L. grossipedia* was collected from Chengde of Hebei Province, China (116°10′ E, 41°28′ N). All samples were morphologically identified and preserved in 95% ethanol at −20 °C for DNA extraction. All specimens and vouchers (No. LGPHECD01-11) were deposited in the State Key Laboratory of Palaeobiology and Stratigraphy, Nanjing Institute of Geology and Palaeontology, Chinese Academy of Sciences, Nanjing, China. Total DNA was extracted using the DNeasy tissue kit (Qiagen, Hilden, Germany) following the manufacturer’s instructions.

### 2.2. PCR Amplification, Sequencing, Sequence Assembly, and Gene Annotation

The mitochondrial genome was amplified by Polymerase Chain Reaction (PCR) using 14 primer pairs (Supplementary Table S1). Amplification reactions and sequencing were performed according to the previously described method [38], and assembling of mtDNA fragments, annotation of mitogenome, and comparison followed the procedure of Sun and Cheng [39]. Overlapped mtDNA fragments were assembled into contigs using BioEdit 7.0.9.0 [40]. Sequence annotation and accurate boundary determination of PCGs and rRNA genes were first performed by the NCBI’s web interface for BLAST [41] and then by alignment with the homologous genes from other released sequences of Branchiopoda. Miotochondrial tRNA genes and their secondary structures were identified by a combination of MITOS online software [42] and tRNAscan-SE 1.2.1 online software [43]. 

### 2.3. Sequence Alignment and Substitutional Saturation Test

Mitochondrial genomes of 44 relevant taxa were retrieved from GenBank (http://www.ncbi.nlm.nih.gov, accessed on 28 September 2022), together with our newly generated Laevicaudata mitogenome, resulting in a dataset (Supplementary Table S2). The dataset is composed of 42 ingroup species representing 16 families and 3 orders of Branchiopoda: Diplostraca (5 species), Notostraca (3 species), and Anostraca (22 species). *Hutchinsoniella macracantha* Sanders, 1955 (Cephalocarida: Hutchinsoniellidae) and *Squilla biformis* Bigelow, 1891 (Malacostraca: Squillidae) were selected as outgroups. The amino acid sequences of 13 protein-coding genes (PCGs) were aligned using MUSCLE implemented in MEGA X [44]. The corresponding nucleotide sequences of each PCG were aligned by the aligned amino acid sequences implemented in DAMBE 6 [45].

We estimated saturation for each PCG, and for the three codon positions using DAMBE 6 [45], which determined an “index of substitution saturation” (*I*_ss_) based on the notion of entropy in information theory. We excluded partitions or genes which showed significant nucleotide saturation from phylogenetic analyses. The non-synonymous substitution rate (*K_a_*) for each taxon was calculated in comparison with the outgroup using DAMBE 6 [45].

### 2.4. Analyses of Sequence Heterogeneity and Phylogenetic Signal Dissection

We calculated the base composition of each taxon for each PCG and compared the AT% for each gene among the branchiopod species included in this study. The compositional diversity of amino acids of 13 PCGs across branchiopod suborders was obtained by calculating the frequency of four amino acids which were encoded by GC-rich codons (glycine, alanine, arginine and proline; GARP). The homogeneity of substitution pattern (*I_D_* test) for each gene was estimated using a Monte-Carlo method with 1000 replicates implemented in MEGA X [44]. The null hypothesis that sequences have evolved with the same pattern of substitution was rejected at α < 0.01. We also evaluated the compositional heterogeneity in each of the 13 mitochondrial proteins separately by performing posterior predictive analysis (PPA) with the global test statistic as implemented in PhyloBayes 4.1c [46].

A significant conflict between the branchiopod phylogenies is the placement of Laevicaudata. To resolve the deep relationship between Laevicaudata and Onychocaudata and to address the sources of deep phylogenetic conflict, we divided taxa into four clades: (1) Anostraca; (2) Notostraca; (3) Laevicaudata; and (4) Onychocaudata. 

Three methods for reducing the nonphylogenetic signals were conducted: (1) exclusion of the genes with the most strongly deviating composition according to the AT% of *L. grossipedia*; (2) exclusion of the proteins with a significant model violation according to posterior predictive analysis (PPA); and (3) removal of fast-evolving sites. To evaluate the key phylogenetic splits and visualize the phylogenetic content of datasets, we conducted four-cluster likelihood mapping analyses (FcLM) using both nucleotide and amino acid datasets as implemented in TreePuzzle v5.3 [47]. We preferred the topology of the currently accepted relationships within Branchiopoda: (((Laevicaudata, Onychocaudata), Notostraca), Anostraca).

### 2.5. Phylogenetic Analysis

Five datasets were used for phylogenetic analyses: (1) 13 protein-coding genes without the third codon positions (the PCG12 matrix; 7587 bp); (2) amino acid sequences of 13 PCGs (the PAA matrix; 3796 aa); (3) a concatenated nucleotide sequence alignment of the first and the second codon positions of six PCGs including *cox1*, *cox2*, *cox3*, *cytb*, *atp6* and *nad3* (the Pnuc6 matix; 3464 bp); (4) a concatenated amino acid sequence alignment of seven PCGs except *atp8*, *nad2*, *nad4*, *nad4l, nad5* and *nad6* (the Paa7 matrix; 2043 aa); (5) a concatenated amino acid sequence alignment removing fast-evolving sites from Paa7 matrix (the Paas7 matrix; 1094 aa). 

#### 2.5.1. Phylogenetic Analyses under Site-Homogeneous Models

In order to compare the results of phylogenetic inference from different evolutionary models, phylogenetic analyses of four datasets (PCG12, PAA, Pnuc6 and Paa7) were first carried out under site-homogeneous models implemented in RAxML 2.2.3 [48] for maximum likelihood inference (ML) and Bayesian inference using MrBayes 3.2 [49]. Because highly heterogeneous sequence divergence was present across branchiopod clades, and applying standard homogenous models might prompt inaccurate inferences, we did not apply this method to the Paas7 matrix. The best-fit model for the nucleotide dataset (Pnuc6) according to the Akaike Information Criterion (AIC) was determined using jModelTest version 0.1.1 [50], and ProtTest 3 [51] was used for the amino acid dataset (Paa7). The best selected partition schemes and models of two datasets were listed in Supplementary Table S3. For the ML analyses, branch support of two datasets was estimated using the rapid bootstrap method in RAxML with 1000 replicates. Analyses with the software MrBayes were conducted in two simultaneous runs, each with four chains, for 10 million generations, and trees being sampled every 1000 generations. The first 25% were discarded as burn-in, and the remaining trees were used to calculate Bayesian posterior probability (BPP) values. Values of the Potential Scale Reduction Factor (PSRF) approaching 1.0 suggested that the runs reached convergence.

#### 2.5.2. Phylogenetic Analyses under Site-Heterogeneous Models

Substitution saturation is recognized as one of the primary obstacles for deep phylogenetic inference, and removing sites that have experienced multiple substitutions would make for erratic phylogenetic estimates [52]. In order to correctly retrieve the phylogenetic signals of pattern-heterogeneity from mitogenomic sequence data, we performed Bayesian inference analyses under the site-heterogeneous model CAT + GTR for three datasets (Pnuc6, Paa7, and Paas7), as implemented in PhyloBayes 4.1c [46]. Two independent chains of 5000 cycles were run for each analysis, with one point every five samples. The initial 1000 trees sampled in each MCMC run were discarded as burn-in after checking for convergence using bpcomp (max_diff < 0.3). The 50% majority-rule consensus tree and the associated posterior probabilities (PPs) were then computed using all chains. 

Bayesian cross-validation [53] was used to compare the fit of site-homogeneous (LG and GTR) and site-heterogeneous (CAT-mtREV and CAT-GTR) models as implemented in PhyloBayes 4.1c [46]. Ten replicates were conducted, 1100 sampling cycles were run and the first 100 samples were discarded as burn-in. Fast-evolving sites for Paa7 matrix were identified using the discrete gamma rate category to which they belong using TreePuzzle v5.3 [47], and the sites belonging to the most rapidly evolving gamma category were removed.

## 3. Results

### 3.1. Characteristics of L. grossipedia Mitogenome

The complete mitogenome of *L. grossipedia* is 15,023 bp in length (GenBank accession number: OP746066). This is the first completely sequenced mitogenome in the order Laevicaudata. All of the 37 typical animal mitochondrial genes were identified, consisting of 13 PCGs, 22 tRNAs, two mitochondrial ribosomal RNAs (rrnS and rrnL) and a putative control region (Table 1). Twenty-three genes were encoded by the majority strand (J-strand) and fourteen by the minority strand (N-strand). Gene arrangement of the branchiopod mitogenomes was considered to be rather well-conserved, although several events of translocation, inversion, tandem duplication and random loss have occurred [33,34]. We found two gene rearrangement phenomena in the mitogenome of *L. grossipedia*: (1) the local inversion of *trnI,* and (2) the remote inversion of *trnL_1_.* The latter observed at the *nad1*–*rrnL* junction was the dominant gene rearrangement event in Branchiopoda. In addition to the control region, the mitogenome of *L. grossipedia* had 181 bp of intergenic nucleotides in 13 different locations, with intergenic spacer lengths ranging from 1 to 63 bp. The longest intergenic spacer was located between *trnG* and *nad3* (Table 1). In the *L. grossipedia* mitogenome, ATN codons initiated all PCGs. Six PCGs used TAA/TAG as the termination codons, while truncated termination codons (T) was observed in the other seven genes (Table 1). 

For *L. grossipedia,* the AT content of the complete genome, PCGs, rRNA, tRNA and control region were greater than those of *Leptestheria brevirostris* Barnard, 1924, 75%, 73.6%, 76.8%, 75.8% and 81%, respectively (Table 2). The highest AT content occurs in the third codon position of PCGs (84.3%). *Atp8* had a very high AT content (83.7%), while the lowest AT content was found in *cox1* (66.8%). The AT contents of *L. grossipedia* were the highest when compared with other species of Branchiopoda, showing an obvious AT mutation bias. In most branchiopods, the mitogenome has a positive AT-skew and negative GC-skew [37,39]. *L. grossipedia* and *Leptestheria brevirostris* exhibited a similar pattern (Table 2). The nucleotide skew statistics for the mitochondrial genomes of *L. grossipedia* analysed in the present study also indicated the following: (1) the AT-skew of all PCGs was negative (−0.32 ~ −0.07); (2) the GC-skew was positive and the AT-skew of each PCG on the minority strand was negative, whereas both the GC-skew and AT-skew of each PCG on the majority strand were negative (except *cox1*) (Table 2).

### 3.2. Levels of Substitutional Saturation and Heterogeneous Sequence Divergence within Branchiopod Mitogenomes

The third codon positions were saturated for all genes, and about half of the first and second codon position also showed significant levels of saturation (Table S4). Therefore, they were not considered for further phylogenetic analyses.

The value of *K_a_* was low for Notostraca (0.24~0.25), Spinicaudata (0.26~0.27) and Cladocera (0.25~0.27), but generally higher for Anostraca (0.32~0.38) and Laevicaudata (0.32 ± 0.01), which suggested that Anostraca and Laevicaudata had relatively higher evolutionary rates among Branchiopoda. We analysed the compositional heterogeneity of both the nucleotides and amino acids of 13 PCGs across branchiopod suborders. There was considerable variation in the AT content of mitogenomes within branchiopods (47.8%~83.7%), and Laevicaudata had the highest AT% by a high margin (Table 3). There was considerable variation in GC-encoding GARP amino acids of the mitochondrial genome within Branchiopoda (range: 14.37%~18.78%; mean: 17.66%; standard deviation: 1.24), and Laevicaudata had the lowest GARP%. Our observation showed a high degree of compositional heterogeneity among branchiopod mitogenomes in both nucleotide and amino acid level, which led to systematic error in phylogenetic analyses [11,54,55,56]. To test the homogeneity of substitution pattern, we made 903 pairwise comparisons to calculate the *I_D_*. When we compared the concatenated 13 PCGs, 719 comparisons had a statistically significant heterogeneous substitution pattern, suggesting that the substitution pattern evolved multiple times. The *I_D_* test on each 13 PCGs also suggested a high level of variation in the substitution patterns among different genes (Table 3). The null hypothesis that sequences have evolved with the same pattern of substitution was rejected (α < 0.01), although a correlation was observed between the level of variation in the substitution patterns and the gene lengths.

### 3.3. Phylogenetic Analyses Using Standard Homogeneous Models

**Figure 1:**
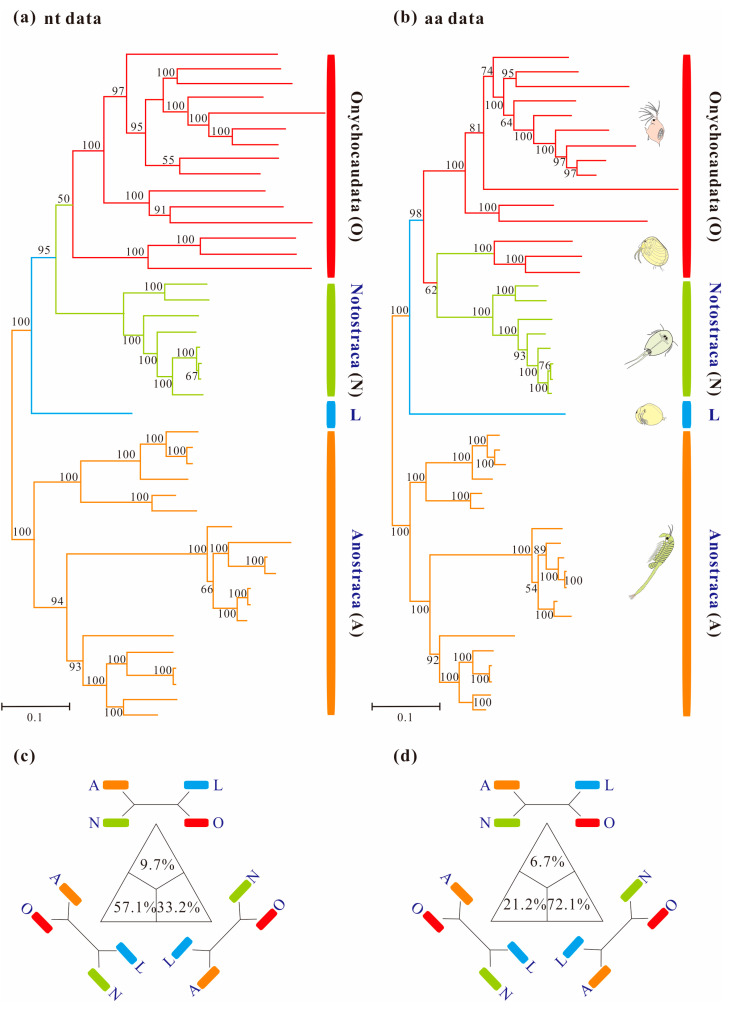
Maximum-likelihood phylograms of Branchiopoda based on concatenation of 13 mitochondrial genes under site-homogeneous models obtained with RAxML: (**a**) PCG12 matrix and (**b**) PAA matrix. Major groups are labeled and each group is indicated with a representative line drawing. Nodal supports are bootstrap values. The trees of Branchiopoda between the nucleotide and amino acid datasets exhibit incongruence by the monophyly of Onychocaudata. (**c**) and (**d**) are results based on the PCG12 and PAA datasets respectively using the four-cluster likelihood mapping analyses. For each matrix, the sequences are divided into four clades: (**a**) Anostraca; (**b**) Notostraca; (**c**) Laevicaudata; (**d**) Onychocaudata. The corners of the triangles represent the three alternative topologies.

**Figure 2:**
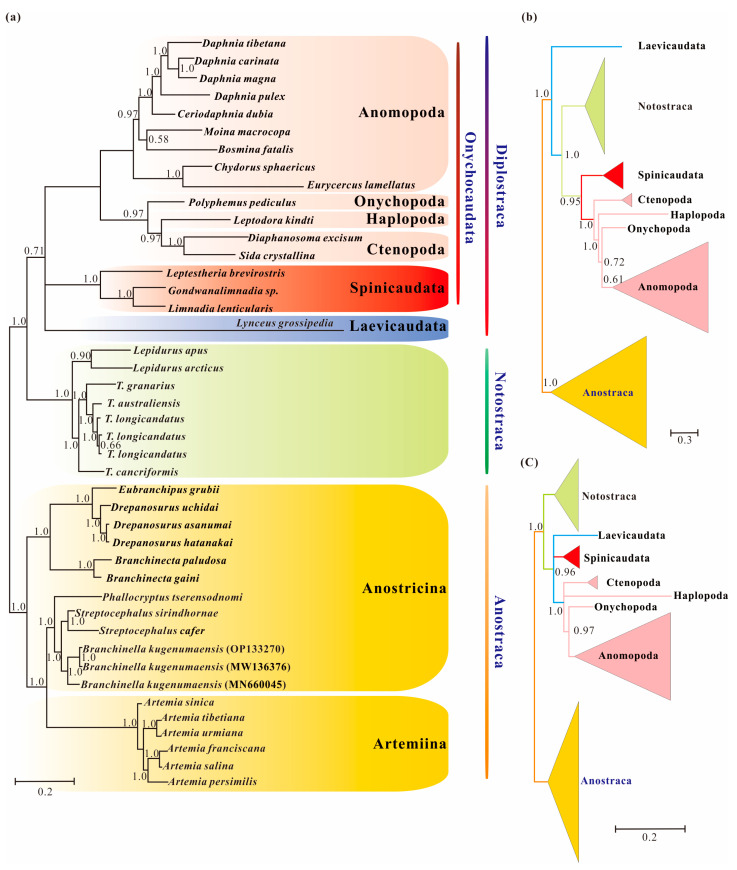
Branchiopod phylogenies inferred from the datasets of Pnuc6, Paa7 and Paas7 under the site-heterogeneity models obtained with PhyloBayes: (**a**) Bayesian tree from the dataset of Pnuc6; (**b**) Bayesian tree from the dataset of Paa7; (**c**) Bayesian tree from the dataset of Paas7. The Bayesian trees based on the datasets of Paa7 and Paas7 are shown in b and c as a schematic version with some lineages collapsed for clarity. Supports at nodes are Bayesian posterior probabilities.

Homogeneous analyses of either nt or aa data yielded maximal support for the monophyly of Anostraca and Phyllopoda (ML_nt&aa_-BS = 100%; Figure 1). Phyllopoda included four major groups (Cladocera, Spinicaudata, Laevicaudata and Notostraca), and Laevicaudata was resolved as a sister to the remaining phyllopods. However, the relationships among these four groups differed based on different datasets: monophyletic Notostraca was resolved as a sister to Onychocaudata when inferences were drawn from nucleotide data (ML_nt&aa_-BS = 95%; Figure 2a), whereas Notostraca occupied a sister position to Spinicaudata based on the amino acid data (ML_nt&aa_-BS = 62%; Figure 2b), which were consistent with the four-cluster likelihood mapping analyses (nt: 57.1% and aa: 72.1%, Figure 2c,d). These results, based on site-homogeneous analyses, were congruent with previous mitogenomes analyses [37], but not consistent with the currently accepted sister group relationship between Laevicaudata and Onychocaudata [26,29,33]. The conflict was not resolved by the Bayesian and ML analyses under site-homogenous models with a partitioning scheme for both Pnuc6 and Paa7 datasets, each matrix supporting similar trees (Figure S1) to those presented in Figure 1.

### 3.4. Reducing Compositional Heterogeneity in Sequence Data

Phylogenetic analyses of the individual mitochondrial genes and proteins and PPA test demonstrated that both the nucleotide composition of all 13 PCGs and the amino acid composition of six among the 13 mitochondrial proteins violated the assumptions of the CAT model (Table 4), indicating that compositional bias was usually a genome-wide phenomenon [54].

The sequence heterogeneity analysis showed that Laevicaudata exhibited significantly higher heterogeneity than the other branchiopods. Laevicaudata being resolved as the basal clade of Phyllopoda with high support was most likely due to artifactual phylogenetic inferences, probably resulting from the high degree of heterogeneity. If we excluded Laevicaudata, no inferences can be made about the relationships of this taxon. Accordingly, we applied three exclusive uses of homoplastic sites methods to reduce the potential impact of compositional heterogeneity on phylogenetic inference. Using the concatenated sequences of the 13 PCGs or 13 mitochondrial proteins for phylogenetic inference was proven to be not impactful in reconciling model misspecification (Table 4). When only seven mitochondrial proteins or six PCGs with the lowest *Z* scores were used for the phylogenetic analyses, the *Z* scores were reduced, but the compositional heterogeneity was still significant (*Z* = 7.27 for 7 proteins; *Z* = 5.00 for 6 PCGs). However, when only the Paas7 matrix was used for phylogenetic analyses, the CAT model was no longer violated (*p* = 0.09, *Z* = −1.48). Therefore, the compositional heterogeneity in the concatenated sequences of mitochondrial proteins could be reduced to a degree that the CAT model was no longer violated.

### 3.5. Phylogenetic Results under Heterogeneous Model

Nonstationary heterogeneous composition models, which account for compositional heterogeneity among lineages, have been manifested to control systematic errors in tree reconstruction [10,57]. The results of Bayesian cross-validation tests showed that: (1) The CAT-GTR+Γ_4_ mixture model offered a better fit to the data compared with GTR+Γ_4_ (2ΔlnL = 430 ± 48), and (2) the CAT-mtREV+Γ_4_ mixture model was better, compared with LG + Γ_4_ (2ΔlnL = 16086 ± 1546).

Bayesian inference from the Pnuc6 dataset under a site-heterogeneous model recovered the monophyly of Diplostraca, but with low probability (BI_nt_-PP = 0.71; Figure 2a). The result indicated that the high support for Laevicaudata as the earliest branch of Phyllopoda under site-homogeneous models was partly due to among-lineage compositional bias. In contrast, the analysis of the Paa7 matrix using the CAT-mtREV+Γ_4_ mixture model model did not support the monophyly of Diplostraca, and the result supported Laevicaudata as a sister to the rest of Phyllopoda (Figure 2b), as did site-composition homogeneous models. When we removed fast-evolving sites (46.45%) from Paa7 matrix, the monophyly of Diplostraca was recovered with high support (BI_nt_-PP = 0.96; Figure 2c), confirming that the removal of the fast-evolving positions increased the ratio of phylogenetic to non-phylogenetic signal [58]. This observation also implied that compositional heterogeneity and fast-evolving positions in the amino acid datasets were two sources of phylogenetic artifacts and nonstationary heterogeneous composition model showed significant improvements over site-homogenous models in the phylogenetic reconstruction.

## 4. Discussion

### 4.1. Pervasiveness of Phylogenetic Conflicts

**Figure 3:**
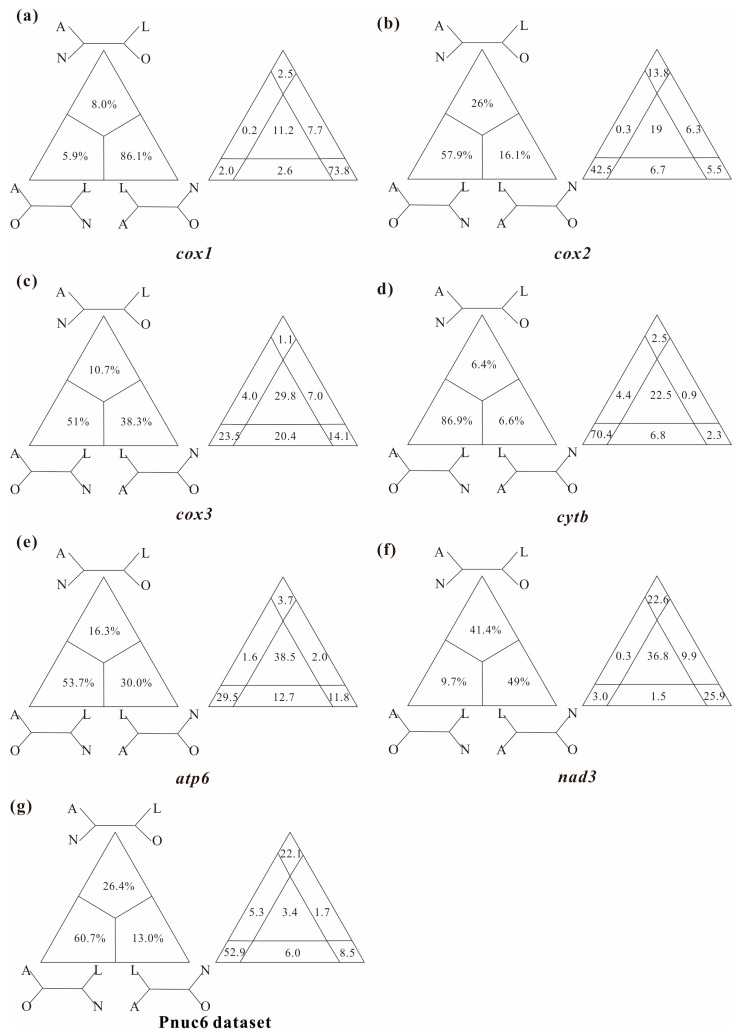
Conflict visualization using four-cluster likelihood mapping analyses as implemented in Treepuzzle based on the nucleotide sequence alignment of the first and the second codon positions of *cox1* (**a**), *cox2* (**b**), *cox3* (**c**), *cytb* (**d**), *atp6* (**e**), *nad3* (**f**), and Pnuc6 dataset (**g**). Quartet proportions (in %) are mapped on a 2D-simplex graph supporting different quartet topologies on the major phylogenetic relationships within Branchiopoda.

**Figure 4:**
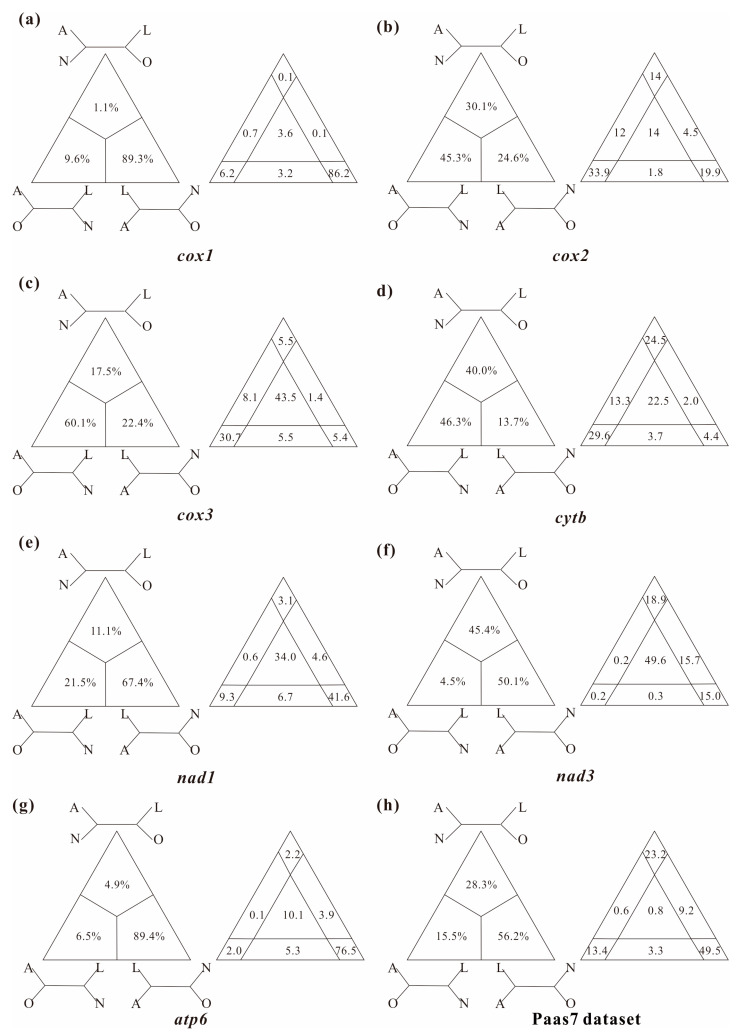
Conflict visualization using four-cluster likelihood mapping analyses as implemented in Treepuzzle based on the amino acid sequences of *cox1* (**a**), *cox2* (**b**), *cox3* (**c**), *cytb* (**d**), *nad1* (**e**), *nad3* (**f**), atp6 (**g**), and Paas7 dataset (**h**). Quartet proportions (in %) are mapped on a 2D-simplex graph supporting different quartet topologies on the major phylogenetic relationships within Branchiopoda.

In this study, several datasets were utilized to test basal relationships of Diplostraca and to compare the results of phylogenetic inference from different evolutionary models. Nevertheless, the standard phylogenetic methods consistently failed to uncover the correct phylogeny (Figure 1 and Figure S1). High nodal supports concealed the pervasiveness of phylogenetic conflicts. The non-monophyly of Diplostraca supported by these analyses was an artifactual result overturning a key relationship supported by morphological cladistic studies [26,27,28,31] and phylogenomic analyses [29,30,32,59]. The monophyly of Diplostraca was supported based on the list of supporting synapomorphies, such as bivalved carapaces in adults, larvae with small and budlike first antennae, highly modified first male thoracopod pair for clasping females and trunk limb exopods in adults with long dorsal lobes [31]. Furthermore, the recovery of the monophyly of Diplostraca through the exclusive use of homoplastic sites and the application of site-heterogeneous models (Figure 2a) confirmed that the non-monophyly of Diplostraca was an artifact. In FcLM analyses based on nucleotide sequences, the majority of quartets supported Notostraca as the closest relatives of Laevicaudata (Figure 3). This is congruent with part of the current results (Figure 2a and Figure S1a). In FcLM analyses based on amino acid sequences, the majority of quartets supported Notostraca as the closest relatives of Onychocaudata (Figure 4). This is again congruent with part of our results (Figure 2b and Figure S1b). Using amino acid sequences or the removal of fast-evolving sites was considered an efficient approach to reduce systematic errors and to resolve deep relationships [60,61,62]. However, the quartet puzzling analysis plotted the probability of the preferred: (((Laevicaudata, Onychocaudata), Notostraca) topology, Anostraca) and the probabilities only ranged from 1.1% to 26.4% (Figure 3 and Figure 4). Measurement of phyologenetic signal showed 0.8% of unresolved quartets and 13.1% of partly resolved quartets presented in the Paas7 matrix (excluding 46.45% sites), and quartet support for preferred topology was still low (23.2%). These results suggested that the phylogenetic signal for a deep relationship between Laevicaudata and Onychocaudata was always weak and differed among amino acid datasets (Figure 3). These findings could be explained by the decay of the phylogenetic signal or a limited signal in the mitogenomic sequences. The limitations of mitogenomes applied in deep phylogeny of Arthropod have already been pointed out [63] and emphasized [1,4,5,6,64]. When the non-phylogenetic signal was higher than the phylogenetic signal due to mutational saturation, high AT-content, parasitic life-styles or explosive radiation events, considerable systematically erroneous relationships were recovered [6]. Our analyses confirmed these conclusions.

### 4.2. Heterogeneity and Tree Topology

The comparisons of AT% between Laevicaudata and Spinicaudata (Table 2), the *I_D_* test (Table 3) and the PPA test (Table 4) demonstrated a high degree of compositional heterogeneity among branchiopod mitogenomes at both the nucleotide and amino acid levels, which could bias the phylogenetic inference. The CAT+GTR model used in Bayesian inference analyses, implemented in PhyloBayes 4.1c [46], was chosen for its superiority in accommodating site-heterogeneous patterns of molecular evolution [11,12]. However, the Bayesian analysis under the site-heterogeneous model for the amino acid dataset (Paa7 matrix) recovered a topology almost identical to the phylogenetic analysis under the site-homologous model for the amino acid dataset of the PAA matrix (Figure 1b and Figure 2b). This suggested that the homoplasy in amino acid datasets was not only due to compositional heterogeneity considered in site-heterogeneous model. When we applied amino acid recoding to our datasets (removing fast-evolving sites from Paa7 matrix; the Paas7 matrix) combined with the site-heterogeneous model, the monophyly of Diplostraca was recovered correctly (Figure 2c). Our study demonstrated that removing fast-evolving sites could be an effective method to overcome the among-site rate heterogeneity from nonstationarity [65,66]. Although phylogenetic resolution to the monophyly of Diplostraca was improved when we applied a variety of strategies to reduce the effects of saturation and heterogeneity, the deep relationships within Diplostraca were not fully resolved.

The sum of these analyses suggested that the phylogenetic resolution of Diplostraca using mitogenomes was trapped by conflicting phylogenetic signals existing across different genes, which in turn was aggravated by compositional heterogeneity and among-site rate heterogeneity. The phylogenetic signal and the potential influence of non-phylogenetic signal should be independently evaluated when mitogenomic datasets were applied in deep phylogeny.

## 5. Conclusions

In this study, we extensively dissected the potential sources of non-phylogenetic signal that resulted in high support but incorrect phylogenies when mitogenomes were applied in deep phylogeny. We identified significant compositional heterogeneity in both the nucleotide and amino acid datasets. Phylogenetic analyses under site-homogeneous models suggested that topological conflict at the base of Phyllopoda were retained across all datasets, even with the exclusion of the genes with the most strongly deviating compositions. Bayesian inference under the site-heterogeneous CAT-GTR+Γ_4_ mixture model using the nucleotide dataset (Pnuc6) recovered the monophyly of Diplostraca. However, it is limited for the amino acid dataset, regardless of minimization of model violation. Although slow-evolving sites of the amino acid dataset (Paas7) under the site-heterogeneous model revealed the monophyly of Diplostraca with high support, the deep relationships among Laevicaudata, Spinicaudata and Cladocera were not fully resolved, which demonstrated systematic conflicts in phylogenetic signal. The results of FcLM analyses confirmed the systematic conflicts and revealed that the phylogenetic signal for deep relationship between Laevicaudata and Onychocaudata was significantly weaker than the nonphylogenetic signal across all datasets. Future analyses including the mitogenomes of the other laevicauatan species are needed to achieve a more complete understanding of the evolutionary history of Diplostraca by identifying more basal branches.

## Figures and Tables

**Table 1:** Annotation of the mitochondrial genome of *L. grossipedia*.

Gene	GenBank Position no.	Size (nts)	Strand ^a^	Start Codon	Stop Codon	Anticodon	IGN ^b^
*trnI*	1–64	64	-			GAT	46
*trnQ*	111–179	69	-			TTG	9
*trnM*	189–253	65	+			CAT	12
*nd2*	266–1216	951	+	ATA	TAG		−2
*trnW*	1215–1277	63	+			TCA	−1
*trnC*	1277–1339	63	-			GCA	0
*trnY*	1340–1405	66	-			GTA	−5
*cox1*	1401–2939	1539	+	ATA	TAA		2
*trnL1*-CUN	2942–3006	65	+			TAG	1
*trnL2*-UUR	3008–3069	62	+			TAA	19
*cox2*	3089–3770	682	+	ATT	T		1
*trnK*	3771–3835	65	+			CTT	0
*trnD*	3836–3898	63	+			GTC	0
*atp8*	3899–4060	162	+	ATT	TAA		−4
*atp6*	4057–4720	664	+	ATA	T		0
*cox3*	4721–5508	788	+	ATG	T		−1
*trnG*	5509–5569	61	+			TCC	63
*nd3*	5633–5990	358	+	ATA	T		3
*trnA*	5991–6052	62	+			TGC	14
*trnR*	6067–6126	60	+			TCG	−3
*trnN*	6124–6187	64	+			GTT	0
*trnS1*-AGN	6188–6244	57	+			GCT	0
*trnE*	6245–6307	63	+			TTC	0
*trnF*	6308–6369	62	-			GAA	0
*nd5*	6370–8041	1672	-	ATT	T		0
*trnH*	8042–8103	62	-			GTG	0
*nd4*	8104–9403	1300	-	ATG	T		−1
*nd4L*	9403–9690	295	-	ATG	TAA		5
*trnT*	9696–9757	62	+			TGT	0
*trnP*	9758–9820	63	-			TGG	2
*nd6*	9823–10,303	481	+	ATT	T		0
*cytb*	10,304–11,434	1131	+	ATG	TAA		−2
*trnS2*-UCN	11,433–11,499	67	+			TGA	4
*nd1*	11,504–12,415	912	-	ATT	TAA		0
*rrnL*	12,416–13,738	1323	-				0
*trnV*	13,739–13,806	68	-			TAC	0
*rrnS*	13,807–14,586	780	-				0
Control region	14,589–15,023	437	+				0

^a^ Plus strand (+)/mius strand (-); ^b^ Number of intergenic nucleotides. Numbers of IGN indicate non-coding nucleotides between genes (positive values) or gene overlap (negative values).

**Table 2:** Nucleotide composition and skewness levels of *L. grossipedia* (Laevicaudata)/*Leptestheria brevirostris* (Spinicaudata).

Regions	Nucleotide Composition (%)					AT-Skew	GC-Skew
	T (U)	C	A	G	A + T		
Whole genome	36.8/31.9	15.3/22.4	38.1/26.5	9.9/19.2	75.0/59.5	0.02/−0.09	−0.21/−0.08
PCGs	43.8/35.4	12.5/22.6	29.8/22.0	13.8/20.0	73.6/57.4	−0.19/−0.23	0.05/−0.06
1st codon position	35.4/29.0	12.6/20.5	32.0/25.6	20.0/25.1	67.4/54.4	−0.05/−0.06	0.24/0.10
2nd codon position	48.6/44.0	16.9/22.2	20.5/17.3	14.0/16.5	69.2/61.4	−0.41/−0.43	−0.10/−0.15
3rd codon position	47.4/33.0	8.1/25.1	37.0/23.0	7.6/18.5	84.3/56.0	−0.12/−0.19	−0.03/−0.15
rRNA	39.3/27.3	7.6/16.3	37.5/34.8	15.7/21.53	76.8/62.1	−0.02/0.12	0.35/0.14
tRNA	36.7/31.3	10.7/16.6	39.1/30.1	13.5/22.1	75.8/61.3	0.03/−0.02	0.12/0.14
*atp6*	42.0/35.1	16.9/25.2	31.5/20.1	9.6/19.5	73.5/55.3	−0.14/−0.27	−0.28/−0.13
*atp8*	45.6/33.3	12.2/28.8	38.1/23.1	4.1/14.7	83.7/56.4	−0.09/−0.18	−0.5/−0.32
*cox1*	39.2/33.5	16.7/23.7	27.6/20.5	16.5/22.2	66.8/54.1	−0.17/−0.24	−0.01/−0.03
*cox2*	38.2/30.8	16.6/24.7	32.9/22.9	12.3/21.6	71.1/53.7	−0.07/−0.15	−0.15/−0.07
*cox3*	39.5/37.1	17.1/22.3	29.8/19.6	13.7/21.0	69.2/56.7	−0.14/−0.31	0.11/−0.03
*cytb*	39.5/34.3	16.6/24.4	31.6/20.6	12.4/20.7	71.0/54.9	−0.11/−0.25	−0.14/−0.08
*nad1*	48.7/36.4	8.5/18.6	26.2/24.1	16.6/20.8	74.9/60.6	−0.30/−0.20	0.32/0.06
*nad2*	46.3/40.0	11.4/27.1	31.8/17.6	10.4/15.4	78.2/57.6	−0.19/−0.39	−0.05/−0.28
*nad3*	43.2/40.2	15.3/19.9	30.2/18.8	11.3/21.1	73.4/59.0	−0.18/−0.36	−0.15/0.03
*nad4*	49.8/36.5	7.4/21.1	27.9/22.5	14.9/20.8	77.8/59.0	−0.28/−0.24	0.34/0.02
*nad4L*	52.2/34.3	3.4/19.9	26.8/24.9	17.5/20.9	79.0/59.3	−0.32/−0.16	0.67/0.02
*nad5*	44.5/32.3	8.1/20.6	31.8/26.6	15.6/20.5	76.2/58.8	−0.17/0.10	0.31/0.00
*nad6*	45.8/39.1	13.1/25.7	34.2/20.1	6.9/15.1	80.0/59.2	−0.15/−0.32	−0.31/−0.26

**Table 3:** *I_D_* test summary and AT% on individual mitochondrial genes.

Gene	Number of Comparisons with Significant Heterogeneity	Proportion of Significant Heterogeneity (%)	AT% ^a^			
			Max	Mean	Min	LG ^b^
*nd2*	554	61.4	78.2	68.6	57.2	78.2
*cox1*	588	65.1	66.8	61.7	54.1	66.8
*cox2*	368	40.8	71.1	64.2	53.7	71.1
*atp8*	123	13.6	83.7	70.2	47.8	83.7
*atp6*	435	48.2	73.5	64.8	54.6	73.5
*cox3*	597	66.1	69.2	62.3	51.2	69.2
*nd3*	350	38.8	76.8	68.7	56.8	73.5
*nd5*	710	78.6	76.2	66.5	54.8	76.2
*nd4*	692	76.6	77.8	67.0	55.1	77.8
*nd4l*	318	35.2	79.0	68.8	59.3	79.0
*nd6*	334	37.0	80.0	69.9	55.7	80.0
*cytb*	569	63.0	71.0	62.9	54.9	71.0
*nd1*	598	66.2	74.9	65.6	56.9	74.9

Note: For each gene, a total of 903 pairwise comparisons are made and shown. The null hypothesis that sequences have evolved with the same pattern of substitution is rejected (α < 0.01). AT% ^a^: AT% of each 13 PCGs of Branchiopoda; LG ^b^: AT% of each 13 PCGs of *L. grossipedia.*

**Table 4:** Results of Posterior Predictive Test on individual mitochondrial genes.

Gene/Datasets	Nucleotide Data Sets			Amino Acid Data Sets		
	*Z* Score	*p* Score	NDT *	*Z* Score	*p* Score	NDT *
*nd2*	6.42	0.00	31	3.50	0.00	18
*cox1*	5.20	0.02	32	0.37	0.24	6
*cox2*	4.82	0.03	18	0.01	0.42	0
*atp8*	5.67	0.00	11	2.11	0.03	14
*atp6*	4.67	0.00	25	0.26	0.30	4
*cox3*	4.31	0.02	31	−0.89	0.81	2
*nd3*	6.05	0.01	20	0.41	0.29	3
*nd5*	13.10	0.00	33	5.79	0.00	16
*nd4*	6.81	0.00	39	4.34	0.00	10
*nd4l*	7.12	0.00	26	2.04	0.05	5
*nd6*	5.67	0.01	24	2.15	0.05	5
*cytb*	6.59	0.00	26	0.92	0.19	3
*nd1*	5.13	0.00	35	1.28	0.09	7
13 PCGs	5.60	0.00	39	13.18	0.00	33

NDT *: Number of taxa with significantly deviating composition.

## Data Availability

All gene sequence data are available from GenBank (http://www.ncbi.nlm.nih.gov, accessed on 28 September 2022).

## Supplementary Materials

The following supporting information can be downloaded at: https://www.mdpi.com/article/10.3390/cimb45020054/s1, Figure S1: Phylogenetic trees of Branchiopoda (colour-coded) obtained with RAxML and MrBayes inferred from the datasets of Pnuc6 (a) and Paa7 (b). Supports at nodes are ML bootstrap values and Bayesian posterior probabilities; Table S1: List of primer combinations used to amplify the mitochondrial genome of *L*. *grossipedia*; Table S2: Details of species and mitogenomes of Branchiopoda used in this study; Table S3: Partition schemes and best-fitting models for phylogenetic analyses; Table S4: Results of the test for substitution saturation. The references [67,68,69,70,71,72,73,74,75,76,77,78,79,80,81,82,83,84,85,86,87] are cited in the Supplementary Materials.

## Abbreviations

*atp6*: ATPase subunit 6; *atp8*: ATPase subunit 8; *cox1*: Cytochrome c oxidase subunit I; *cox2*: Cytochrome c oxidase subunit II; *cox3*: Cytochrome c oxidase subunit III; *cytb*: mitochondria-encoded cytochrome B; *nad1*: NADH dehydrogenase subunit I; *nad2*: NADH dehydrogenase subunit II; *nad3*: NADH dehydrogenase subunit III; *nad4*: NADH dehydrogenase subunit IV; *nad4L*: NADH dehydrogenase subunit 4L; *nad5*: NADH dehydrogenase subunit V; *nad6*: NADH dehydrogenase subunit VI; tRNA: mitochondrial transfer RNA; *rrnS*: mitochondrial small ribosomal subunit RNA; *rrnL*: mitochondrial large ribosomal subunit RNA.

## References

[1] Cameron S.L., Lambkin C.L., Barker S.C., Whiting M.F. (2007). A mitochondrial genome phylogeny of Diptera: Whole genome sequence data accurately resolve relationships over broad timescales with high precision. Syst. Entomol..

[2] Harrison G.L., McLenachan P.A., Phillips M.J., Slack K.E., Cooper A., Penny D. (2004). Four new avian mitochondrial genomes help get to basic evolutionary questions in the late Cretaceous. Mol. Biol. Evol..

[3] Phillips M.J., Penny D. (2003). The root of the mammalian tree inferred from whole mitochondrial genomes. Mol. Phylogenet. Evol..

[4] Cook C.E., Yue Q., Akam M. (2005). Mitochondrial genomes suggest that hexapods and crustaceans are mutually paraphyletic. Proc. Biol. Sci..

[5] Rota-Stabelli O., Kayal E., Gleeson D., Daub J., Boore J.L., Telford M.J., Pisani D., Blaxter M., Lavrov D.V. (2010). Ecdysozoan mitogenomics: Evidence for a common origin of the legged invertebrates, the Panarthropoda. Genome Biol. Evol..

[6] Talavera G., Vila R. (2011). What is the phylogenetic signal limit from mitogenomes? The reconciliation between mitochondrial and nuclear data in the Insecta class phylogeny. BMC Evol. Biol..

[7] Delsuc F., Brinkmann H., Chourrout D., Philippe H. (2006). Tunicates and not cephalochordates are the closest living relatives of vertebrates. Nature.

[8] Rodríguez-Ezpeleta N., Brinkmann H., Roure B., Lartillot N., Lang B.F., Philippe H. (2007). Detecting and Overcoming Systematic Errors in Genome-Scale Phylogenies. Syst. Biol..

[9] Philippe H., Brinkmann H., Lavrov D.V., Littlewood D.T.J., Manuel M., Wörheide G., Baurain D. (2011). Resolving difficult phylogenetic questions: Why more sequences are not enough. PLoS Biol..

[10] Foster P.G. (2004). Modeling compositional heterogeneity. Syst. Biol..

[11] Lartillot N., Philippe H. (2008). Improvement of molecular phylogenetic inference and the phylogeny of Bilateria. Philos. Trans. R. Soc. B.

[12] Lartillot N., Philippe H. (2004). A Bayesian mixture model for across-site heterogeneities in the amino-acid replacement process. Mol. Biol. Evol..

[13] Ho S.Y.W., Jermiin L.S. (2004). Tracing the decay of the historical signal in biological sequence data. Syst. Biol..

[14] Pagel M., Meade A. (2004). A phylogenetic mixture model for detecting pattern-heterogeneity in gene sequence or character-state data. Syst. Biol..

[15] Martin J.W., Belk D. (1988). Review of the clam shrimp family Lynceidae Stebbing, 1902 (Branchiopoda: Conchostraca), in the Americas. J. Crustac. Biol..

[16] Timms B.V. (2013). A revision of the Australian species of *Lynceus* Müller, 1776 (Crustacea: Branchiopoda: Laevicaudata: Lynceidae). Zootaxa.

[17] Rogers D.C., Olesen J. (2016). Laevicaudata catalogus (Crustacea: Branchiopoda): An overview of diversity and terminology. Arthropod Syst. Phylogeny.

[18] Sigvardt Z.M.S., Rogers D.C., De los Ríos P., Palero F., Olesen J. (2019). First molecular phylogeny of Laevicaudata (Crustacea: Branchiopoda) with description of a new species of *Lynceus* from Chile and an updated key to species in the Americas. Invertebr. Syst..

[19] Olesen J., Martin J.W., Martin J.W., Olesen J., Høeg J.T. (2014). Laevicaudata. Atlas of Crustacean Larvae.

[20] Pessacq P., Epele L.B., Rogers D.C. (2011). A new species of *Lynceus* (Crustacea: Branchiopoda: Laevicaudata) from Patagonia, with comments on laevicaudatan systematics. Zootaxa.

[21] Rogers D.C., Olesen J., Martin J.W. (2015). A new possibly parthenogenic species of *Lynceus* from Canada (Crustacea: Branchiopoda: Laevicaudata), with key to the Nearctic female Laevicaudata. Sci. Pap. Nat. Hist. Mus. Univ. Kansas.

[22] Olesen J., Pöllabauer C., Sigvardt Z.M.S., Rogers D.C. (2016). A new species of *Lynceus* Müller, 1776 from New Caledonia (Crustacea: Branchiopoda: Laevicaudata) from dolines, with remarks on zoogeography. Eur. J. Taxon..

[23] Shu S., Sigvardt Z.M.S., Chen X., Olesen J., Rogers D.C., Sanoamuang L. (2019). *Lynceus amplopedia* sp. nov., a new laevicaudatan clam shrimp with asymmetrically modified thoracopods from Yunnan, China (Crustacea: Branchiopoda). Zool. Stud..

[24] Sigvardt Z.M.S., Shu S., Alonso M., Ventura M., Sanoamuang L., Rogers D.C., Palero F., Olesen J. (2020). A new Northeast Asian *Lynceus* (Crustacea: Branchiopoda: Laevicaudata) with uniquely modified thoracopods and an evaluation of DNA barcoding for clam shrimp species identification. Nauplius.

[25] Sigvardt Z.M.S., Olesen J., Rogers D.C., Timms B., Mlambo M., Rabet N., Palero F. (2021). Multilocus phylogenetics of smooth clam shrimps (Branchiopoda, Laevicaudata). Zool. Scr..

[26] Richter S., Olesen J., Wheeler W.C. (2007). Phylogeny of Branchiopoda (Crustacea) based on a combined analysis of morphological data and six molecular loci. Cladistics.

[27] Olesen J. (2009). Phylogeny of Branchiopoda (Crustacea)—Character evolution and contribution of uniquely preserved fossils. Arthropod Syst. Phylogeny.

[28] Olesen J., Richter S. (2013). Onychocaudata (Branchiopoda: Diplostraca), a new high-level taxon in branchiopod systematics. J. Crustac. Biol..

[29] Schwentner M., Richter S., Rogers D.C., Giribet G. (2018). Tetraconatan phylogeny with special focus on Malacostraca and Branchiopoda: Highlighting the strength of taxon-specific matrices in phylogenomics. Proc. Biol. Sci..

[30] Uozumi T., Ishiwata K., Grygier M.J., Sanoamuang L.O., Su Z. (2021). Three nuclear protein-coding genes corroborate a recent phylogenomic model of the Branchiopoda (Crustacea) and provide estimates of the divergence times of the major branchiopodan taxa. Genes Genet. Syst..

[31] Olesen J. (2007). Monophyly and phylogeny of Branchiopoda, with focus on morphology and homologies of branchiopod phyllopodous limbs. J. Crustac. Biol..

[32] Lozano-Fernandez J., Giacomelli M., Fleming J.F., Chen A., Vinther J., Thomsen P.F., Glenner H., Palero F., Legg D.A., Iliffe T.M. (2019). Pancrustacean evolution illuminated by taxon-rich genomic-scale data sets with an expanded remipede sampling. Genome Biol. Evol..

[33] Luchetti A., Forni G., Skaist A.M., Wheelan S.J., Mantovani B. (2019). Mitochondrial genome diversity and evolution in Branchiopoda (Crustacea). Zool. Lett..

[34] Castellucci F., Luchetti A., Mantovani B. (2022). Exploring mitogenome evolution in Branchiopoda (Crustacea) lineages reveals gene order rearrangements in Cladocera. Sci. Rep..

[35] Kitano T., Sato H., Takahashi N., Igarashi S., Hatanaka Y., Igarashi K., Umetsu K. (2022). Complete mitochondrial genomes of three fairy shrimps from snowmelt pools in Japan. BMC Zool..

[36] Sun X., Cheng J. (2022). Comparative Mitogenomic Analyses and New Insights into the Phylogeny of Thamnocephalidae (Branchiopoda: Anostraca). Genes.

[37] Xu S., Han B., Martínez A., Schwentner M., Fontaneto D., Dumont H.J., Kotov A.A. (2021). Mitogenomics of Cladocera (Branchiopoda): Marked gene order rearrangements and independent predation roots. Mol. Phylogenet. Evol..

[38] Sun X. (2021). Divergence across the mitogenomes of *Branchinella kugenumaensis* (Anostraca: Thamnocephalidae) with implications for species delimitation. Mitochondrial DNA Part B.

[39] Sun X., Cheng J. (2019). Characterization of the complete mitochondrial genome of Chinese *Triops granarius* and implications for species delimitation. Int. J. Biol. Macromol..

[40] Hall T.A. (1999). BioEdit: A user-friendly biological sequence alignment editor and analysis program for Windows 95/98/NT. Nucleic Acids Symp. Ser..

[41] BLAST. https://blast.ncbi.nlm.nih.gov/Blast.cgi.

[42] Bernt M., Donath A., Jühling F., Externbrink F., Florentz C., Fritzsch G., Pütz J., Middendorf M., Stadler P.F. (2013). MITOS: Improved de novo metazoan mitochondrial genome annotation. Mol. Phylogenet. Evol..

[43] Schattner P., Brooks A.N., Lowe T.M. (2005). The tRNAscan-SE, snoscan and snoGPS web servers for the detection of tRNAs and snoRNAs. Nucleic Acids Res..

[44] Kumar S., Stecher G., Li M., Knyaz C., Tamura K. (2018). MEGA X: Molecular evolutionary genetics analysis across computing platforms. Mol. Biol. Evol..

[45] Xia X. (2017). DAMBE6: New tools for microbial genomics, phylogenetics, and molecular evolution. J. Hered..

[46] Lartillot N., Lepage T., Blanquart S. (2009). PhyloBayes 3: A Bayesian software package for phylogenetic reconstruction and molecular dating. Bioinformatics.

[47] Schmidt H.A., Strimmer K., Vingron M., von Haeseler A. (2002). TREE-PUZZLE: Maximum likelihood phylogenetic analysis using quartets and parallel computing. Bioinformatics.

[48] Stamatakis A. (2006). RAxML-VI-HPC: Maximum likelihood-based phylogenetic analyses with thousands of taxa and mixed models. Bioinformatics.

[49] Ronquist F., Teslenko M., van der Mark P., Ayres D.L., Darling A., Höhna S., Huelsenbeck J.P. (2012). MrBayes 3.2: Efficient Bayesian phylogenetic inference and model choice across a large model space. Syst. Biol..

[50] Posada D. (2008). jModelTest: Phylogenetic model averaging. Mol. Biol. Evol..

[51] Darriba D., Taboada G.L., Doallo R., Posada D. (2011). ProtTest 3: Fast selection of best-fit models of protein evolution. Bioinformatics.

[52] Fan L., Wu D., Goremykin V., Xiao J., Xu Y., Garg S., Zhang C., Martin W.F., Zhu R. (2020). Phylogenetic analyses with systematic taxon sampling show that mitochondria branch within Alphaproteobacteria. Nat. Ecol. Evol..

[53] Stone M. (1974). Cross-validatory choice and assessment of statistical predictions. J. R. Stat. Soc. Ser. B Methodol..

[54] Nesnidal M.P., Helmkampf M., Bruchhaus I., Hausdorf B. (2010). Compositional heterogeneity and phylogenomic inference of metazoan relationships. Mol. Biol. Evol..

[55] Rota-Stabelli O., Pisani D. (2013). Serine codon-usage bias in deep phylogenomics: Pancrustacean relationships as a case study. Syst. Biol..

[56] Liu Y., Song F., Jiang P., Wilson J.-J., Cai W., Li H. (2018). Compositional heterogeneity in true bug mitochondrial phylogenomics. Mol. Phylogenet. Evol..

[57] Phillips M.J., Delsuc F., Penny D. (2004). Genome-scale phylogeny and the detection of systematic biases. Mol. Biol. Evol..

[58] Brochier C., Philippe H. (2002). Phylogeny: A nonhyperthermophilic ancestor for Bacteria. Nature.

[59] Regier J.C., Shultz J.W., Zwick A., Hussey A., Ball B., Wetzer R., Martin J.W., Cunningham C.W. (2010). Arthropod relationships revealed by phylogenomic analysis of nuclear protein-coding sequences. Nature.

[60] Brinkmann H., Philippe H. (1999). Archaea sister group of Bacteria? Indications from tree reconstruction artifacts in ancient phylogenies. Mol. Biol. Evol..

[61] Ruiz-Trillo I., Riutort M., Littlewood D.T., Herniou E.A., Baguna J. (1999). Acoel flatworms: Earliest extant bilaterian Metazoans, not members of Platyhelminthes. Science.

[62] Martijn J., Vosseberg J., Guy L., Offre P., Ettema T.J.G. (2018). Deep mitochondrial origin outside the sampled alphaproteobacteria. Nature.

[63] Curole J., Kocher T. (1999). Mitogenomics: Digging deeper with complete mitochondrial genomes. Trends Ecol. Evol..

[64] Nardi F., Spinsanti G., Boore J.L., Carapelli A., Dallai R., Frati F. (2003). Hexapod origins: Monophyletic or paraphyletic?. Science.

[65] Cameron S.L., Miller K.B., D’Haese C.A., Whiting M.F., Barker S.C. (2004). Mitochondrial genome data alone are not enough to unambiguously resolve the relationships of Entognatha, Insecta and Crustacea *sensu lato* (Athropoda). Cladistics.

[66] Sheffield N.C., Song H., Cameron S.L., Whiting M.F. (2009). Nonstationary evolution and compositional heterogeneity in beetle mitochondrial phylogenomics. Syst. Biol..

[67] Crease T.J. (1999). The complete sequence of the mitochondrial genome of *Daphnia pulex* (Cladocera: Crustacea). Gene.

[68] Geng X., Cheng R., Cheng D., Zhang H. (2016). The complete mitochondrial DNA genome of Chinese *Daphnia carinata* (Clasocera: Daphniidae). Mitochondrial DNA Part B.

[69] Cheng R., Deng B., Wang Y., Geng X., Li J., Zhang X., Peng S., Deng D., Zhang H. (2016). Complete mitochondrial genome sequence of *Daphnia magna* (Crustacea: Cladocera) from Huaihe in China. J. Lake Sci..

[70] Yang R., Chen Y. (2020). The complete mitochondrial genome of the freshwater fairy shrimp *Branchinella kugenumaensis* Ishikawa 1894 (Crustacea: Anostraca: Thamnocephalidae). Mitochondrial DNA Part B.

[71] Fan Y., Lu B., Yang J. (2016). The complete mitogenome of the fairy shrimp *Phallocryptus tserensodnomi* (Crustacea: Anostraca: Thamnocephalidae). Mitochondrial DNA Part A.

[72] Tladi M., Dalu T., Rogers D.C., Nyamukondiwa C., Emami-Khoyi A., Oliver J.C., Teske P.R., Wasserman R.J. (2020). The complete mitogenome of an undescribed clam shrimp of the genus *Gondwanalimnadia* (Branchiopoda: Spinicaudata), from a temporary wetland in Central District, Botswana. Mitochondrial DNA Part B.

[73] Liu X., Li H., Jermnak U., Yang J. (2016). The complete mitogenome of the freshwater fairy shrimp *Streptocephalus sirindhornae* (Crustacea: Anostraca: Streptocephalidae). Mitochondrial DNA Part A.

[74] Jo E., Kim J., Ko Y., Kim S., Kang S. (2022). The complete mitochondrial genome of the Antarctic fairy shrimp *Branchinecta gaini* Daday, 1910 (Branchiopoda, Anostraca, Branchinectidae). Biodivers. Data J..

[75] Perez M., Valverde J., Batuecas B., Amat F., Marco R., Garesse R. (1994). Speciation in the *Artemia* genus: Mitochondrial DNA analysis of bisexual and parthenogenetic brine shrimps. J. Mol. Evol..

[76] Zhang H., Luo Q., Sun J., Liu F., Wu G., Yu J., Wang W. (2013). Mitochondrial genome sequences of *Artemia tibetiana* and *Artemia urmiana*: Assessing molecular changes for high plateau adaptation. Sci. China Life Sci..

[77] Asem A., Li W., Wang P., Eimanifar A., Shen C., de Vos S., van Stappen G. (2019). The complete mitochondrial genome of *Artemia sinica* Cai, 1989 (Crustacea: Anostraca) using next-generation sequencing. Mitochondrial DNA Part B.

[78] Deji G., Zhang C., Sui L., Han X. (2021). The complete mitochondrial genome of *Artemia salina* Leach, 1819 (Crustacea: Anostraca). Mitochondrial DNA Part B.

[79] Han X., Tashi L., Sui L., Wang G., Deji G., Zhang C. (2022). The complete mitochondrial genome of *Artemia persimilis* Piccinelli and Prosdocimi, 1968 (Crustacea: Anostraca). Mitochondrial DNA Part B.

[80] Bellec L., Debruyne R., Utge J., Rabet N. (2019). The first complete mitochondrial genome of *Limnadia lenticularis* (Branchiopoda, Spinicaudata), with new insights on its phylogeography and on the taxonomy of the genus. Hydrobiologia.

[81] Emami-Khoyi A., Tladi M., Dalu T., Teske P.R., van Vuuren B.J., Rogers D.C., Nyamukondiwa C., Wasserman R.J. (2021). The complete mitogenome of *Leptestheria brevirostris* Barnard, 1924, a rock pool clam shrimp (Branchiopoda: Spinicaudata) from Central District, Botswana. Mitochondrial DNA Part B.

[82] Umetsu K., Iwabuchi N., Yuasa I., Saitou N., Clark P.F., Boxshall G., Osawa M., Igarashi K. (2002). Complete mitochondrial DNA sequence of a tadpole shrimp (*Triops cancriformis*) and analysis of museum samples. Electrophoresis.

[83] Gan H., Tan M., Austin C.M. (2016). The complete mitogenome of the Australian tadpole shrimp *Triops australiensis* (Spencer and Hall, 1895) (Crustacea: Branchiopoda: Notostraca). Mitochondrial DNA.

[84] Horn R.L., Cowley D.E. (2014). Evolutionary relationships within *Triops* (Branchiopoda: Notostraca) using complete mitochondrial genomes. J. Crustac. Biol..

[85] Ryu J., Hwang U. (2010). Complete mitochondrial genome of the longtail tadpole shrimp *Triops longicaudatus* (Crustacea, Branchiopoda, Notostraca). Mitochondrial DNA.

[86] Lavrov D.V., Brown W.M., Boore J.L. (2004). Phylogenetic position of the Pentastomida and (pan)crustacean relationships. Proc. R. Soc. Lond. B.

[87] Koga C., Rouse G.W. (2021). Mitogenomics and the phylogeny of mantis shrimp (Crustacea: Stomatopoda). Diversity.

