# Supplementary material for: Conflicts in Mitochondrial Phylogenomics of Branchiopoda, with the First Complete Mitogenome of Laevicaudata (Crustacea: Branchiopoda)
Source: Curr Issues Mol Biol. 2023 Jan 18;45(2):820–37. doi: 10.3390/cimb45020054 (PMC9955068; doi:10.3390/cimb45020054)
Supplement: Supplementary file 1 [file cimb-45-00054-s001.zip › Figure S1 Caption.pdf]

Figure S1. Phylogenetic trees of Branchiopoda (colour-coded) obtained with RAxML and MrBayes inferred from the datasets of Pnuc6 (a) and Paa7 (b). Supports at nodes were ML bootstrap values and Bayesian posterior probabilities.
