# Supplementary material for: Conflicts in Mitochondrial Phylogenomics of Branchiopoda, with the First Complete Mitogenome of Laevicaudata (Crustacea: Branchiopoda)
Source: Curr Issues Mol Biol. 2023 Jan 18;45(2):820–37. doi: 10.3390/cimb45020054 (PMC9955068; doi:10.3390/cimb45020054)
Supplement: Supplementary file 1 [file cimb-45-00054-s001.zip › Table S1 Primers of Lynceus grossipedia.pdf]

**Table S1.** List of primer combinations used to amplify the mitochondrial genome of *Lynceus grossipedia*

| Fragment | Region | Primer pair | Primer sequence (5′–3′) |
|---|---|---|---|
| LGS 1 | *cox1* | LGS 1F | CTGATATAGCTTTTCCTCG |
| | | LGS 1R | AATTTTAATACCAGTAGGTAC |
| LGS 2 | *cox1-atp6* | LGS 2F | TGGGATAATCTATGCTATAT |
| | | LGS 2R | AACTCAAAAAAGGGGTAAAAT |
| LGS 3 | *atp6* | LGS 3F | CTATTTCAATCATTTGACCCC |
| | | LGS 3R | GCAGCTAATCGCACGGCTAA |
| LGS 4 | *atp6-cox3* | LGS 4F | AAAACTGTAGAAATATTAGCCC |
| | | LGS 4R | AAAGGCTCAGAAGAAGGAAAC |
| LGS 5 | *cox3* | LGS 5F | TCAATGATGACGAGATATTAC |
| | | LGS 5R | CAAACAACATCAACAAAATGTC |
| LGS 6 | *cox3-nad4* | LGS 6F | CTTATTGGTTCAACATTTTTA |
| | | LGS 6R | AGGTTGATACCCCCAACCAA |
| LGS 7 | *Nad4* | LGS 7F | GGGGATACAACCTGAACGG |
| | | LGS 7R | TACAGGAGCTTCAACATGA |
| LGS8 | *nad4-cytb* | LGS 8F | ATGATTTTAGCTGGTATTTT |
| | | LGS 8R | GAAAAAAATCGATTTAAGGT |
| LGS9 | *cytb* | LGS 9F | GGTTATGTTCTCCCTTGAG |
| | | LGS 9R | CATTCTGGTTGAATGTGAGT |
| LGS10 | *cytb-16S* | LGS 10F | GTTTATATTATGGGTCATATA |
| | | LGS 10R | CACCGGTCTGAACTCAGATC |
| LGS11 | *16S* | LGS 11F | AGACCGGTGTGAGCCAGG |
| | | LGS 11R | TCACCGGGCAGGTATTACCT |
| LGS12 | *16S-12S* | LGS 12F | TTGTCTTTTAATTGAAGACTG |
| | | LGS 12R | ACTATCATTTACTGAACAGG |
| LGS13 | *12S* | LGS 13F | ATCAGTTTAGAGGAACCTG |
| | | LGS 13R | CTACTATGTTACGACTTATCTC |
| LGS14 | *12S-cox1* | LGS 14F | CTAGGATTAGATACCCTATTA |
| | | LGS 14R | TATTACTAGATAAAGGAGGG |
