# Supplementary material for: Conflicts in Mitochondrial Phylogenomics of Branchiopoda, with the First Complete Mitogenome of Laevicaudata (Crustacea: Branchiopoda)
Source: Curr Issues Mol Biol. 2023 Jan 18;45(2):820–37. doi: 10.3390/cimb45020054 (PMC9955068; doi:10.3390/cimb45020054)
Supplement: Supplementary file 1 [file cimb-45-00054-s001.zip › Table S2 Details of species and mitogenomes of Branchiopoda.pdf]

Table S2. Details of species and mitogenomes of Branchiopoda used in this study.

Family	Genus	Species	Length (bp)	Accession ID	Reference
Chydoridae	*Chydorus*	*Chydorus sphaericus* (O.F.Müller, 1776)	15,959	MT872704	No reference
Eurycercidae	*Eurycercus*	*Eurycercus lamellatus* (O.F.Müller, 1776)	15,775	MT872696	No reference
Daphniidae	*Daphnia*	*Daphnia tibetana* (Sars, 1903)	16,196	MW981579	No reference
		Daphnia pulex O.F.Müller, 1776	15,333	AF117817	67
		Daphnia carinata King, 1853	15,245	NC_028152	68
		Daphnia magna Straus, 1820	14,948	NC_026914	69
	Ceriodaphnia	*Ceriodaphnia dubia* Richard, 1894	14,628	LC705054	No reference
Moinidae	*Moina*	*Moina macrocopa* (Straus, 1820)	16,072	MZ982313	No reference
Bosminidae	*Bosmina*	*Bosmina fatalis* Burckhardt, 1924	15,209	MW770308	No reference
Sididae	*Sida*	*Sida crystallina* (O.F.Müller, 1776)	16,072	MT872684	No reference
	Diaphanosoma	*Diaphanosoma excisum* Sars 1885	17,615	MW476927	No reference
Leptodoridae	*Leptodora*	*Leptodora kindti* (Focke, 1844)	15,860	MT872700	No reference
Polyphemidae	*Polyphemus*	*Polyphemus pediculus* (Linnaeus, 1761)	13,456	MT862416	No reference
Lynceidae	*Lynceus*	*Lynceus grossipedia* Sigvardt et al., 2020	15,121	OP746066	This study
Thamnocephalidae	*Branchinella*	*Branchinella kugenumaensis* (Ishikawa, 1895) (Japan)	14,123	MW136376	38
		Branchinella kugenumaensis (Ishikawa, 1895) (Zhejiang, China)	15,127	MN660045	70
		Branchinella kugenumaensis (Ishikawa, 1895) (Jiangsu, China)	14,126	OP133270	36
	Phallocryptus	*Phallocryptus*	16,493	KP273592	71

		tserensodnomi Alonso & Ventura, 2013			
Streptocephalidae	*Streptocephalus*	*Streptocephalus cafer* (Lovén, 1847)	17,020	MN720104	72
		Streptocephalus sirindhornae Sanoamuang, Murugan, Weekers & Dumont, 2000	16,887	NC_026704	73
Chirocephalidae	*Drepanosurus*	*Drepanosurus uchidai* (Kikuchi, 1957)	15,795	LC633442	35
		Drepanosurus hatanakai Takahashi & Hamasaki, in Takahashi et al., 2018	17,006	LC633440	
		Drepanosurus asanumai Takahashi, in Takahashi et al., 2018	17,503	LC633438	
	Eubranchipus	*Eubranchipus grubii* (Dybowski, 1860)	16,328	MT410793	No reference
Branchinectidae	*Branchinecta*	*Branchinecta gaini* Daday, 1910	15,536	MZ265218	74
		Branchinecta paludosa (O. F. Müller 1788)	16,059	MZ853171	No reference
Artemiidae	*Artemia*	*Artemia franciscana* Kellogg, 1906	15,822	NC_001620	75
		Artemia urmiana Gunther, 1899	15,945	NC_021382	76
		Artemia tibetiana Abatzopoulos, Zhang & Sorgeloos, 1998	15,742	NC_021383	76
		Artemia sinica Cai, 1989	15,689	MK069595	77
		Artemia salina (Linneaus, 1758)	15,826	MT495441	78
		Artemia persimilis Piccinelli & Prosdocimi, 1968	15,436	MZ199176	79
Limnadiidae	*Limnadia*	*Limnadia lenticularis* (Linnaeus, 1761)	15,151	MH618637	80
	Gondwanalimnadia	*Gondwanalimnadia* sp.	15,661	MN625703.1	72

	Leptestheria	*Leptestheria brevirostris* Barnard, 1924	15,579	MN548772.1	81
Triopsidae	*Triops*	*Triops cancriformis* (Bosc, 1801)	15,101	NC_004465	82
		Triops australiensis (Spencer & Hall, 1896)	15,125	NC_024439	83
		Triops longicaudatus (LeConte, 1846)	15,060	KM516710	84
		Triops longicaudatus (LeConte, 1846)	15,058	KM516711	85
		Triops longicaudatus (LeConte, 1846)	15,115	GU475465	85
		Triops granarius (Lucas, 1864)	15,121	MF496656	39
	Lepidurus	*Lepidurus apus* (Linnaeus, 1758)	14,754	NC_044646	33
		Lepidurus arcticus (Pallas, 1793)	14,807	NC_044654	
Hutchinsoniellidae	*Hutchinsoniella*	*Hutchinsoniella macracantha* Sanders, 1955	16,491	AY456189	86
Squillidae	*Squilla*	*Squilla biformis* Bigelow, 1891	15,688	MW867305	87
