# Supplementary material for: Conflicts in Mitochondrial Phylogenomics of Branchiopoda, with the First Complete Mitogenome of Laevicaudata (Crustacea: Branchiopoda)
Source: Curr Issues Mol Biol. 2023 Jan 18;45(2):820–37. doi: 10.3390/cimb45020054 (PMC9955068; doi:10.3390/cimb45020054)
Supplement: Supplementary file 1 [file cimb-45-00054-s001.zip › Table S3 Partitions&models.pdf]

Table S3. Partition schemes and best-fitting models for phylogenetic analyses

Datasets		Phylogenetic Reconstruction Approaches	
		Maximum Likelihood Best-fitting model	Bayesian Inference Best-fitting model
Amino acid datasets 43taxa 2,043 sites	*cox1*	mtART+I+G+F	RtREV+I+G+F
	cox2	mtART+I +G+F	mtART+I+G+F
	cox3	mtART+I +G+F	mtREV+I+G+F
	cytb	mtART+I +G +F	mtREV+I+G+F
	atp6	mtART+I +G+F	RtREV+I+G+F
	nad1	mtART+I +G+F	mtREV+I+G
	nad3	mtART+I +G	mtREV+I+G+F
Nucleotide datasets 43taxa 3,464 sites	The 1st codon of *cox1*	SYM+G+I	SYM+G+I
	The 2nd codon of *cox1*	GTR+G+I	GTR+G+I
	The 1st codon of *cox2*	GTR +G+I	GTR+G+I
	The 2nd codon of *cox2*	GTR +G+I	GTR+G+I
	The 1st codon of *cox3*	GTR+G+I	GTR+G+I
	The 2nd codon of *cox3*	GTR+G+I	GTR+G+I
	The 1st codon of *cytb*	GTR+G+I	GTR +G+I
	The 2nd codon of *cytb*	GTR+G+I	GTR+G+I
	The 1st codon of *atp6*	GTR+G+I	GTR+G+I
	The 2nd codon of *atp6*	GTR+G+I	GTR+G+I
	The 1st codon of *nad3*	HKY+G+I	HKY+G+I
	The 2nd codon of *nad3*	GTR+G+I	GTR+G+I
