# Supplementary material for: Conflicts in Mitochondrial Phylogenomics of Branchiopoda, with the First Complete Mitogenome of Laevicaudata (Crustacea: Branchiopoda)
Source: Curr Issues Mol Biol. 2023 Jan 18;45(2):820–37. doi: 10.3390/cimb45020054 (PMC9955068; doi:10.3390/cimb45020054)
Supplement: Supplementary file 1 [file cimb-45-00054-s001.zip › Table S4 substitution saturation test for individual genes.pdf]

Table S4. Results of the test for substitution saturation.

Gene	Partition	*Iss*	*Iss.c*	*p* score	Aligned length	C	V	Pi	S
nd2	*1st*	1.03	0.69	0.00	353	25	326	297	25
	2nd	0.91	0.69	0.00	353	50	301	252	49
cox1	*1st*	0.26	0.70	0.00	514	320	192	168	24
	2nd	0.13	0.70	0.00	514	430	82	66	16
cox2	*1st*	0.42	0.69	0.00	228	91	137	124	13
	2nd	0.22	0.69	0.00	228	145	83	65	18
atp8	*1st*	1.18	1.16	0.43	58	8	49	48	1
	2nd	1.14	1.16	0.92	58	8	49	49	0
atp6	*1st*	0.55	0.69	0.02	225	61	163	150	13
	2nd	0.37	0.69	0.00	225	109	115	101	14
cox3	*1st*	0.37	0.68	0.00	264	113	151	126	25
	2nd	0.25	0.68	0.00	264	79	185	88	97
nd3	*1st*	0.59	0.82	0.01	121	32	85	83	2
	2nd	0.38	0.82	0.00	121	47	70	62	8
nd5	*1st*	0.77	0.71	0.14	582	94	485	459	26
	2nd	0.65	0.71	0.16	582	154	425	378	47
nd4	*1st*	0.86	0.70	0.00	453	81	369	355	14
	2nd	0.75	0.70	0.42	453	129	321	293	28
nd4l	*1st*	1.12	0.77	0.01	114	11	94	89	5
	2nd	1.09	0.77	0.04	114	20	86	72	13
nd6	*1st*	1.04	0.70	0.00	179	11	166	157	7
	2nd	0.99	0.70	0.01	179	36	141	127	12
cytb	*1st*	0.25	0.68	0.00	380	167	212	182	30
	2nd	0.22	0.68	0.00	380	241	138	102	36
nd1	*1st*	0.67	0.68	0.87	320	90	225	205	16
	2nd	0.52	0.68	0.03	320	128	187	149	32

Iss: estimated index of substitution saturation for the data set. *Iss.c*: critical values for the index of substitution saturation. *Iss* > *Iss.c* ($P < 0.05$) indicates saturation. C: conserved sites, V: variable sites Pi: parsimony informative sites, S: singleton sites, 1st: the first codon position, 2nd: the second codon position.
