# Supplementary figures and images for: Conflicts in Mitochondrial Phylogenomics of Branchiopoda, with the First Complete Mitogenome of Laevicaudata (Crustacea: Branchiopoda)
Source: Curr Issues Mol Biol. 2023 Jan 18;45(2):820–37. doi: 10.3390/cimb45020054 (PMC9955068; doi:10.3390/cimb45020054)

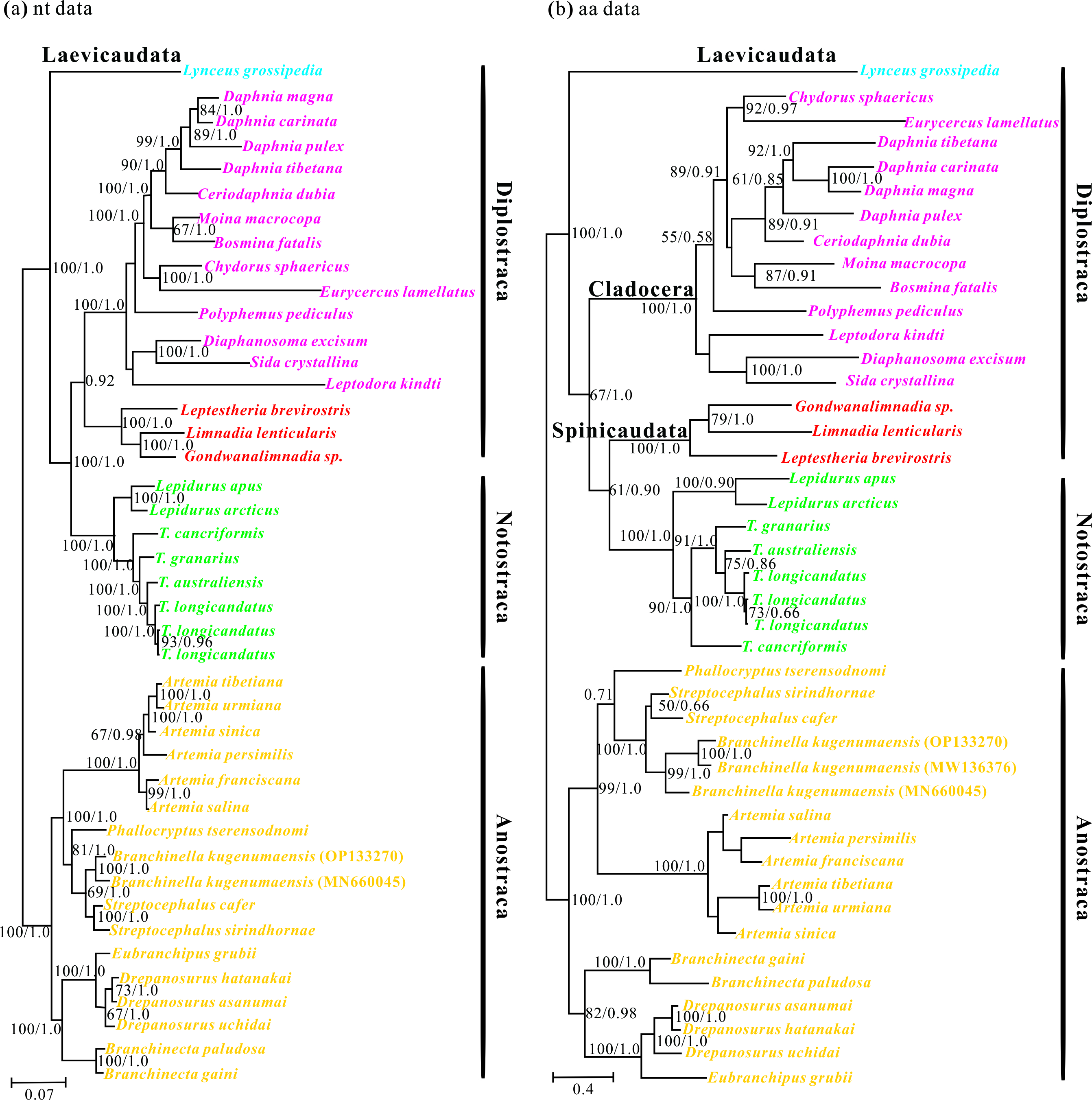

Supplement: Supplementary file 1 [file cimb-45-00054-s001.zip › FIGS1 Phylogenetic trees with RAxML and MrBayes inferred from Pnuc6 and Paa7.jpg]
